# Bloody Amniotic Fluid and Neonatal Outcomes

**DOI:** 10.3390/children10071208

**Published:** 2023-07-12

**Authors:** Hanoch Schreiber, Gal Cohen, Hila Shalev-Ram, Sivan Farladansky-Gershnabel, Omer Weitzner, Tal Biron-Shental, Michal Kovo, Shmuel Arnon, Ofer Markovitch

**Affiliations:** 1 Department of Obstetrics and Gynecology, Meir Medical Center, Kfar Saba 4428163, Israel; hanoch.schreiber@clalit.org.il (H.S.); galco4@clalit.org.il (G.C.); mehila@clalit.org.il (H.S.-R.); sivan.farladansky@clalit.org.il (S.F.-G.); omer.weitzner@clalit.org.il (O.W.); tal.biron-shental@clalit.org.il (T.B.-S.); michal.kovo@clalit.org.il (M.K.); markovitch.ofer@clalit.org.il (O.M.); 2 Faculty of Medicine, Tel Aviv University, Tel Aviv 6329302, Israel; 3 Department of Neonatology, Meir Medical Center, Kfar Saba 4428163, Israel

**Keywords:** bloody amniotic fluid, adverse maternal outcomes, adverse neonatal outcomes, placental abruption

## Abstract

Information on the effect of bloody amniotic fluid during labor at term is scarce. This study assessed risk factors and adverse outcomes in labors with bloody amniotic fluid. During the six years of this study, all nulliparas in our institution, with a trial of labor, were included. Multiple pregnancies and preterm deliveries were excluded. Outcomes were compared between the bloody amniotic fluid group and the clear amniotic fluid group. Overall, 11,252 women were included. Among them, 364 (3.2%) had bloody amniotic fluid and 10,888 (96.7%) had clear amniotic fluid. Women in the bloody amniotic fluid group were characterized by shorter duration of the second stage and higher rate of cesarean section due to non-reassuring fetal heart rate. In addition, there were higher rates of low cord pH (<7.1) and NICU admissions in the bloody amniotic fluid group. In multivariate logistic regression analysis, cesarean delivery, cord blood pH < 7.1, and NICU admission were independently associated with increased odds ratio for bloody amniotic fluid. Bloody amniotic fluid at term is associated with adverse outcomes and must be considered during labor.

## 1. Introduction

The color of amniotic fluid during labor varies and may range from clear, meconium-stained, yellow, to bloody, which can indicate different clinical situations, and accordingly may be associated with adverse maternal and neonatal outcomes. Bloody amniotic fluid (BAF) during labor can be a sign of placental abruption [1,2,3,4,5].

Placental abruption is defined as premature detachment of the placenta from its attachment in the uterus prior to the birth of a fetus. In addition to BAF, other clinical findings of placental abruption include abdominal pain, uterine tenderness, uterine contractions, and non-reassuring fetal heart rate (NRFHR) [6,7]. The incidence of placental abruption during labor varies depending on the population studied and is estimated to occur in about 1% of all pregnancies [8,9,10]. Several factors are associated with placental abruption, including advanced maternal age, hypertensive disorders as chronic hypertension, preeclampsia and gestational hypertension, smoking, previous placental abruptions, trauma, and multiple gestations [11,12,13]. The management depends on the severity of the abruption, maternal and fetal conditions, and the gestational age [14,15,16,17].

BAF should be differentiated from bloodstained mucus during labor. The latter is a normal and expected sign of cervical dilatation and labor progression. It is usually clear or streaked with pink or red blood and is caused by the breakage of maternal blood vessels in the cervix [18]. This mucus plug may be passed when the cervix begins to dilate. BAF, on the other hand, is usually colored by bright red blood [19,20]. Few studies have investigated the outcomes of pregnancies with BAF. Gluck et al. [4] examined the effect of BAF during labor among 317 deliveries. There were higher rates of labor induction, instrumental deliveries, and cesarean deliveries compared to deliveries with clear amniotic fluid. However, BAF was not associated with a difference in neonatal outcomes or in the composite adverse neonatal outcome. Neonatal adverse outcomes can be used as a measure for the quality of the neonatal and maternity care, especially when data are collected objectively in a computerized system [5]. Using a composite outcome of neonates should include morbidity that is associated with long term sequela such as respiratory morbidity, assisted ventilation, hypoglycemia, admission to the NICU, hyperbilirubinemia, cerebral hemorrhage, and convulsions.

Due to the lack of data regarding BAF during labor, the present study examined risk factors for BAF and the effect of that condition on maternal and neonatal outcomes including parameters that were not evaluated in previous work [4].

## 2. Materials and Methods

All nulliparas singleton deliveries in a single tertiary center from January 2014 to October 2020 that underwent trial of labor at 37 weeks or later were included in this retrospective study. Exclusion criteria were deliveries with meconium amniotic fluid, multiple pregnancies, intrauterine fetal demise, and patients who undergo elective cesarean delivery.

The study cohort was divided into labors with BAF diagnosed during labor (BAF group) and labors with clear amniotic fluid (control group). BAF was determined by the obstetrician and was documented in the delivery report. Notably, patients with BAF were diagnosed clinically as possibly having placental abruption. Data were collected from the computerized medical files of the women and the neonates. Maternal baseline characteristics included the maternal age, BMI (kg/m^2^), and gestational age at delivery. The risks factors that were assessed included diagnosis of diabetes mellitus (DM), pre-gestational DM (PGDM), or gestational DM (GDM). Diagnosis was based on either 75 g glucose load or oral 50 g glucose load approach. The oral glucose tolerance test with the 75 g glucose load measures the plasma glucose concentration at fasting and at 1 h and 2 h after glucose administration. One value of blood glucose concentration above the target value at each interval (fasting state: 92, after 1 h: 180, after 2 h: 153 mg/dL) was defined as a positive result. While using the non-fasting oral 50 g glucose load, blood glucose concentration was measured after 1 h. A value of 140 mg/dL blood glucose or higher was defined as positive screening test. In the case of a positive 50 g load test, the 100 g oral glucose tolerance test was conducted, and the blood glucose concentration was measured at fasting and then 1, 2, and 3 h after 100 g glucose administration. Blood glucose concentrations higher than 95,180 and 155,140 mg/dL, respectively, were defined as a positive test. Additional maternal risk factors that were studied included chronic hypertension, gestational hypertension, or preeclampsia that were defined as hypertensive disorders. Labor and delivery characteristics included labor induction, use of epidural anesthesia, duration of the second stage, instrumental delivery, cesarean delivery (CD), CD due to NRFHR, nuchal cord, and true knot.

Maternal and neonatal outcomes included retained placenta, maternal blood transfusion, neonatal birthweight as was recorded in the labor ward, rate of small for gestational age (SGA), as defined by the birthweight 10th percentile based on local growth charts [21], 5 min Apgar score, as was given by the senior pediatrician who attend the delivery, cord blood pH, phototherapy treatment, as indicated by a pre-established nomograms, neonatal intensive care unit (NICU) admission indicated by anticipation of neonatal complications, neonatal hypoglycemia defined by a serum glucose level <40 mg%, respiratory distress with mechanical ventilation indicated by oxygen requirements and blood gases results, cerebral hemorrhage diagnosed by an ultrasound and interpreted by a pediatric radiologist, and convulsions recorded either by an Electroencephalogram (EEG) or Cerebral Functional Monitoring (CFM).

According to departmental protocol, in all deliveries with BAF, a pediatrician must perform an initial assessment of the neonate in the delivery room. Complications that indicated NICU admission included respiratory distress defined either by tachypnea ≥ 60 breaths per minute or dyspnea defined as using accessory muscles during breathing, apnea episode defined as cessation of breath that leads to hypotonia, bradycardia, and desaturation below 80%, or unresponsiveness to tactile stimulus.

### Statistical Analysis

Statistical analyzes were performed with SPSS-28 software (IBM Corp., Armonk, NY, USA). Nominal data were described as numbers and percentages. Continuous variables were described by means and standard deviations. Metric variables were analyzed using the *t* test. Discrete variables were analyzed by the Chi-square test. Statistically significant was defined as a *p*-value < 0.05. A multivariable logistic regression model was used for BAF, adjusted for maternal age, BMI, nulliparity, week of gestation, epidural, HTN/preeclampsia, and induction of labor.

## 3. Results

During the study period, 11,252 nulliparous with a singleton fetus were delivered in our institution and met the inclusion criteria. Among them, 364 (3.2%) women had BAF and 10,888 (96.7%) women had clear amniotic fluid.

### 3.1. Demographic Characteristics

Maternal characteristics of the study groups are presented in Table 1. The BAF group was characterized by older maternal age as compared to the clear amniotic fluid group (29.4 ± 5.1 years vs. 28.3 ± 5.0 years, *p* < 0.001). There were no differences between groups in gestational age at delivery, maternal BMI > 30, DM, smoking, or hypertensive disorders.

### 3.2. Labor Characteristics

Labor characteristics of the study groups are presented in Table 2. The BAF group was characterized by a shorter second stage of labor as compared to the clear amniotic fluid group (114 ± 74 min vs. 103 ± 69 min, *p* = 0.009, respectively). No difference was found in the CD rate between the study groups (53 (14.6%) in the BAF vs. 1389 (12.7%) in the clear amniotic fluid group). However, it was found that the rate of CD due to NRFHR was higher in the BAF group (37 (10.2%) vs. 768, (7%), respectively, *p* = 0.023). No difference was found between the groups in the rate of labor induction, use of epidural anesthesia, intrapartum fever, instrumental deliveries, or umbilical cord complications (true knot or nuchal cord).

### 3.3. Delivery Outcomes

Table 3 presents the delivery outcomes. There were higher rates of cord blood pH < 7.1 (4.4% vs. 1.5%, *p* < 0.001) and neonatal NICU admissions (2.2% vs. 0.9%, *p* = 0.012), among the BAF group as compared to the clear amniotic fluid group. Neonatal birthweights as well as the rate of retained placenta, maternal blood transfusion, SGA, 5 min Apgar score < 7, neonatal hypoglycemia, cerebral hemorrhage, neonatal convulsions, respiratory distress required mechanical ventilation, and use of phototherapy were all similar between the groups.

### 3.4. Multivariable Logistic Regression

To characterize independently variables that are associated with BAF, we used multivariate analysis adjusted for maternal age, week of gestational age, BMI, diabetes, hypertension/preeclampsia, induction of labor, and epidural anesthesia. It was found that BAF was independently associated with CD due to NRFHR and blood cord pH < 7.1 (Table 4).

## 4. Discussion

The current study assessed risk factors and perinatal outcomes of deliveries complicated with BAF at term. We found that BAF was associated with older maternal age and shorter duration of the second stage of labor. Additionally, CD due to NRFHR, cord pH ≤ 7.1, and neonatal NICU admissions were independently associated with BAF.

Although vaginal bleeding and contractions are the classical clinical features of placental abruption, the few studies that examined the association between BAF and adverse outcomes reported inconclusive results. Based on previous studies, it has been found that parity and older maternal age were found as risk factors for placental abruption [20,22,23]. These studies included nulliparous and parous women with different gestational ages. In the current study, only nulliparas were included, and it was found that older maternal age is a risk factor for BAF. However, according to our data, there were no differences in the rate of advanced maternal age (>35 years) between the study groups.

In contrast to earlier studies [23,24,25], the current research failed to find any correlation between smoking and high blood pressure to BAF. Inconclusive results were found in studies that included only placental abruption at term [4,20]. However, it cannot be ruled out that the results in those studies might differ with a larger sample size.

According to the results of the current study, the second stage duration was significantly shorter in cases of BAF compared to those with clear amniotic fluid. A possible mechanism for this phenomenon is related to the activity of thrombin. Thrombin has been shown to induce myometrial contractions [26,27]. In cases of placental abruption early in pregnancy, this may cause preterm labor [28,29]. Similarly, placental abruption at term during labor might shorten the second stage of labor, due to thrombin-induced uterine contractions. As few studies have addressed this point, further research is needed to assess the second stage duration in the presence of BAF.

The results of the current research support those of previous studies that reported that BAF increases the likelihood of CD in cases where NRFHR is diagnosed [10,20], and in these cases the surgery course may be more complicated [30]. Neonatal birth weight was not different between deliveries complicated with BAF at term compared to those that had clear amniotic fluid. In addition, the incidence of SGA infants was similar between the study groups, indicating that BAF is an event that usually is not associated with long term changes in fetal blood supply, and hence the birth weight is similar. Although rate of cord pH < 7.1 was higher in deliveries complicated with BAF at term, this did not result in higher neonatal complications such as respiratory distress syndrome or hypoglycemia as was described by other studies [4,10]. Neonatal hypoglycemia was not different between BAF deliveries and normal amniotic fluid deliveries, and the incidence was around 5% of deliveries. Asymptomatic early neonatal hypoglycemia can be diagnosed after birth, but the long-term neurodevelopmental outcome varied with other morbidities [22]. The most frequent metabolic abnormality seen in newborns is hypoglycemia. A common problem in the treatment of the newborn infant is the screening of at-risk infants and the management of low blood glucose levels in the first hours to days of life. However, there is no a precise description of neonatal hypoglycemia. Current screening standards and management algorithms are based mainly on expert opinion. In cases of BAF, our local policy is to test neonatal glucose levels at 2 h of age when hypoglycemia is most anticipated [3]. The results of this study do not support measuring glucose levels after BAF deliveries. The higher NICU admission in our study as compared to previous study [4] might be a result of a local policy of admitting deliveries complicated with BAF for observation even without neonatal complications or increased morbidity. Another explanation may be related to the relatively large sample size in the current study. pH < 7.1 was significantly higher in deliveries complicated with BAF at term; however, of note is the 5 min Apgar score that was not different between BAF and clear amniotic fluid deliveries (Table 3). The Apgar score has been shown to be a valuable tool for rapid evaluation of the neonate; however, it frequently has a poor correlation with other markers of intrapartum neonatal well-being. It has been observed that Apgar at 5 min, a metric routinely used to identify infants with poor birth outcomes, is a poor descriptor of neonatal morbidity [25]. Combining the Apgar score and measuring the pH of the umbilical cord in high-risk pregnant women may help identify babies who are at risk. Therefore, our study results indicating a cord pH ≤ 7.1 to be independently associated with BAF during labor but not 5-min Apgar score should be evaluated in further studies.

Limitations of this study stem from its retrospective design. In addition, placental pathology was not available, although placental pathology diagnoses abruption only in about a third of the cases. Additionally, the indication for NICU admission was not prospectively designed, and although these indications stated in the method section were clear, it might be that some clinicians were not following them very strictly. Lastly, data regarding fetal or neonatal anemia were not available.

Despite its limitations, this study has several strengths, including the relatively large cohort that was studied and the same management protocol practiced in a single medical center. Furthermore, the detailed neonatal complications evaluated in this study are well documented by a computerized data recording.

## 5. Conclusions

BAF at term seems to be a marker for placental abruption and a bad prognosis. It was found to be a significant risk factor for adverse outcomes including higher rates of CS due to NRFHR, fetal cord pH ≤ 7.1, and NICU admissions. Given that these outcomes were found to be independently associated with BAF, a finding of BAF must be given substantial weight in the labor management decision-making process. Future research is needed regarding managing labor in case of BAF.

## Figures and Tables

**Table 1:** Baseline characteristics.

Characteristic	Amniotic Fluid		*p*-Value
	Bloody (*n* = 364)	Clear (*n* = 10,888)	
Maternal age (years ± SD)	29.36 ± 5.05	28.32 ± 5.03	<0.001
Maternal age > 35 (*n*, %)	36 (9.9)	886 (8.1)	0.23
Gestational age (weeks + days ± SD)	39 + 1 ± 1	39+1 ± 1	0.905
BMI > 30 (kg/m^2^) (*n*, %)	15 (4.1)	653 (6.0)	0.114
Diabetes (*n*, %)	33 (9.1)	1162 (10.7)	0.328
Smoking (*n*, %)	16 (4.4)	517, 4.7	0.756
Hypertension/Preeclampsia (*n*, %)	20 (5.5)	448 (4.1)	0.193
Male fetus (*n*, %)	193 (53.0)	5475 (50.3)	0.303

BMI: body mass index; diabetes includes pre-gestational and gestational diabetes.

**Table 2:** Labor characteristics.

Characteristic	Amniotic Fluid		*p*-Value
	Bloody (*n* = 364)	Clear (*n* = 10,888)	
Labor induction, *n* (%)	120 (32.9)	3379 (31.0)	0.284
Epidural, *n* (%)	295 (81.0)	8840 (81.2)	0.947
Second stage duration, min ± SD	103 ± 69	114 ± 74	0.009
Intrapartum fever, *n* (%)	22 (6.0)	600 (5.5)	0.661
Instrumental delivery, *n* (%)	60 (16.5)	1981 (18.2)	0.405
Cesarean delivery, *n* (%)	53 (14.6)	1389 (12.7)	0.311
Cesarean delivery due to NRFHR, *n* (%)	37 (10.2)	768 (7.0)	0.023
Nuchal cord, *n* (%)	75 (20.6)	2597 (23.8)	0.225
True knot, *n* (%)	1 (0.2)	81 (0.7)	0.313

NRFHR, non-reassuring fetal heart rate.

**Table 3:** Delivery outcomes.

Outcome	Amniotic Fluid		*p*-Value
	Bloody (*n* = 364)	Clear (*n* = 10,888)	
Retained placenta, *n* (%)	20 (5.5)	468 (4.3)	0.27
Maternal blood transfusion, *n* (%)	7, 1.9	181 (1.7)	0.702
Neonatal birthweight, g ± SD	3186 ± 411	3190 ± 417	0.844
Small for gestational age, *n* (%)	43 (11.8)	1210, 11.1	0.676
5 min Apgar < 7, *n* (%)	3 (0.8)	46 (0.4)	0.252
pH < 7.1, *n* (%)	16 (4.4)	166 (1.5)	<0.001
Phototherapy, *n* (%)	17 (4.7)	575 (5.3)	0.504
NICU admission, *n* (%)	8 (2.2)	98 (0.9)	0.012
Neonatal hypoglycemia, *n* (%)	2 (5.5)	67 (6.1)	0.826
Respiratory distress with mechanical ventilation, *n* (%)	2 (0.55)	48 (0.44)	0.805
Cerebral hemorrhage, *n* (%)	0	2 (0.01)	0.791
Convulsions, *n* (%)	0	4 (0.03)	0.708

NICU, neonatal intensive care unit.

**Table 4:** Multivariate analysis model for adverse outcomes associated with bloody amniotic fluid.

Variable	OR	95% Confidence Interval		*p*-Value
		Lower	Upper	
Cesarean delivery due to NRFHR	1.643	1.046	2.582	0.031
pH < 7.1	2.502	1.19	5.262	0.016

A multivariable logistic regression model was applied for bloody amniotic fluid. The model was adjusted for maternal age at delivery, week of gestational age, BMI, diabetes, hypertension/preeclampsia, induction of labor, and epidural anesthesia.
